# Letter to the editor: Strengthening epidemiological surveillance of *Neisseria gonorrhoea *– beyond the detection of cases

**DOI:** 10.2807/1560-7917.ES.2019.24.25.1900355

**Published:** 2019-06-20

**Authors:** Victoria Hernando, Alicia Magistris, Antonio Nicolau, Susana Ramón, Carmen Varela, Marta Ruiz-Algueró, Asunción Diaz

**Affiliations:** 1 HIV Surveillance and Behavioral Monitoring Unit, National Center of Epidemiology, Carlos III Institute of Health, Madrid, Spain; 2 Epidemiology Unit, Insular Center Ibiza-Formentera, Consejería de Salud, Balearic Islands, Spain; 3 Epidemiology Service, Consejería de Salud, Balearic Islands, Spain; 4 Microbiology Laboratory, Can Misses Hospital, IBSALUT, Ibiza, Balearic Islands, Spain

**Keywords:** Spain, sexually transmitted infections, surveillance, epidemiology

**To the editor:** We have read the interesting article by Eyre DW et al. describing a *Neisseria gonorrhoea* (NG) FC428 clone detected in two female cases who developed infection following contact with United Kingdom (UK)-resident men linked to travel to Ibiza, Spain. These cases provide evidence that the FC428 clone’s transmission is occurring within Europe. The widespread distribution of this clone threatens the effectiveness of gonorrhoea treatment [1].

As both cases had contact with the same sexual network in Ibiza, an exhaustive search of other probable NG cases was developed by the national and local public health authorities. During 2018, 82 cases of gonorrhoea were diagnosed in Ibiza; 80% (n = 66) were men with a median age of 29 years (interquartile range (IQR): 25–36) and 20% (n = 16) were women with a median age of 32.5 years (IQR: 27.5–37.5). Forty percent of the cases (n = 33) were foreigners, but none of them were tourists. Among the foreigners, 26 were men, 20 were from Europe (half of which were from Italy) and 12 were from Latin America. The most common clinical presentation was urethritis among men (62/66) and cervicitis among women (15/16).

A positive culture was confirmed in 90% (n = 74) of the cases; 91% (n=60/66) of cultures were positive among men and 87% (14/16) among women. Of the 82 NG isolates tested, 26.8% showed resistance to penicillin (intermediate susceptibility), 2.4% to ciprofloxacin and 11% to both antibiotics. Resistance to ciprofloxacin and levofloxacin was found in 11% (n = 9) of isolated strains, and 15.8% (n = 13) showed additional resistance to penicillin. Two strains showed resistance to ciprofloxacin, levofloxacin and cefuroxime. None of the isolated and tested strains showed resistance to ceftriaxone. In Ibiza there are two hospitals, one public and one private. The notification of gonococcal cases are compulsory in both hospitals. No positive cases were diagnosed in the private hospital in 2018, and all previously described cases were diagnosed in the public hospital.

The incidence rate of gonococcal infection has increased in Spain since 2001. From 2001–13, the annual change was 11.7% (95% confidence interval (CI): 9.6–13.9) and from 2013–17 it was 26.3% (95% CI: 20.7–32.2). In 2017, the Balearic Islands had one of the highest incidence rates in Spain, with 41.79 cases per 100,000 population [2].

In 2011, the first two cases of ceftriaxone-resistant NG were reported in Catalonia, Spain. Both cases were men who have sex with men (MSM). One of the cases was diagnosed with urethral discharge by a general practitioner. He was treated with 100 mg of doxycycline twice a day for 7 days. The other case was his partner and was asymptomatic, with no signs of proctitis or urethritis. He was treated with levofloxacin for 7 days. In both cases, ceftriaxone-resistant NG strains were isolated [3]; these were the third and fourth ceftriaxone-resistant NG strains isolated in Europe and were genetically related to the strain isolated in France [4]. As all cases were MSM, there is strong indication that resistance clones may circulate in this community.

Spain is one of the participating countries in the European Gonococcal Antimicrobial Surveillance Programme (Euro-GASP) [5]. In its last report, published in 2018, cefixime resistance was isolated from 1.6% of the 365 tested isolates submitted from Spain, but no ceftriaxone resistance was identified. Nevertheless, it is necessary to anticipate an emergency situation in which treatment of NG infection can fail. International travel and new technologies such as social media and dating apps can contribute to the spread of new resistance strains that can compromise NG treatment effectiveness. It is therefore important that we reinforce communication among the countries that are facing this global challenge. At the national level, a multidisciplinary approach is needed, so clinicians, microbiologists, epidemiologists and public health authorities should be involved. Spain has developed a national plan against antimicrobial resistance that includes gonococcal infection as a global threat. One of the milestones of this plan is to reinforce the surveillance and control of antimicrobial resistance.
